# Evaluation of a novel nanocrystalline hydroxyapatite powder and a solid hydroxyapatite/Chitosan-Gelatin bioceramic for scaffold preparation used as a bone substitute material

**DOI:** 10.3906/kim-1912-40

**Published:** 2020-08-18

**Authors:** Sharmin RAHMAN, Kazi Hanium MARIA, Mohammad Saif ISHTIAQUE, Arijun NAHAR, Harinarayan DAS, Sheikh Manjura HOQUE

**Affiliations:** 1 Department of Physics, University of Dhaka, Dhaka Bangladesh; 2 Department of Physics, Mawlana Bhashani Science and Technology University, Tangail Bangladesh; 3 Department of Physics, University of Barisal, Barisal Bangladesh; 4 Materials Science Division, Atomic Energy Centre, Dhaka Bangladesh

**Keywords:** Hydroxyapatite, chitosan, gelatin, FTIR, MTT-assay

## Abstract

Artificially fabricated hydroxyapatite (HAP) shows excellent biocompatibility with various kinds of cells and tissues which makes it an ideal candidate for a bone substitute material. In this study, hydroxyapatite nanoparticles have been prepared by using the wet chemical precipitation method using calcium nitrate tetra-hydrate [Ca(NO_3_)_2_.4H_2_O] and di-ammonium hydrogen phosphate [(NH_4_)_2_ HPO_4_] as precursors. The composite scaffolds have been prepared by a freeze-drying method with hydroxyapatite, chitosan, and gelatin which form a 3D network of interconnected pores. Glutaraldehyde solution has been used in the scaffolds to crosslink the amino groups (|NH_2_) of gelatin with the aldehyde groups (|CHO) of chitosan. The X-ray diffraction (XRD) performed on different scaffolds indicates that the incorporation of a certain amount of hydroxyapatite has no influence on the chitosan/gelatin network and at the same time, the organic matrix does not affect the crystallinity of hydroxyapatite. Transmission electron microscope (TEM) images show the needle-like crystal structure of hydroxyapatite nanoparticle. Scanning Electron Microscope (SEM) analysis shows an interconnected porous network in the scaffold where HAP nanoparticles are found to be dispersed in the biopolymer matrix. Fourier transforms infrared spectroscopy (FTIR) confirms the presence of hydroxyl group (OH^-^) , phosphate group (PO^3-^_4_) , carbonate group (CO^2-^_3_) , imine group (C=N), etc. TGA reveals the thermal stability of the scaffolds. The cytotoxicity of the scaffolds is examined qualitatively by VERO (animal cell) cell and quantitatively by MTTassay. The MTT-assay suggests keeping the weight percentage of glutaraldehyde solution lower than 0.2%. The result found from this study demonstrated that a proper bone replacing scaffold can be made up by controlling the amount of hydroxyapatite, gelatin, and chitosan which will be biocompatible, biodegradable, and biofriendly for any living organism.

## 1. Introduction

Bone tissue engineering requires both the principles of medical and engineering to develop a suitable scaffold to grow on a cell successfully [1–2]. Defected or damaged tissues can be reconstructed by the scaffolds which contain bioactive agents. Human bone is inorganic-organic nanocomposite which is a combination of ceramic and polymer [3]. The growth of the bone mineral is controlled by the organic portion of the bone where the inorganic part influences the mechanical strength [4]. The most challenging part of this process is to imitate the extracellular matrix (ECM) of bone and to disperse the bioactive ceramic phase homogeneously in the prepared matrix [5–6]. The requirement of the high surface area, porosity, biocompatibility, biodegradability is an important approach to generate the cells into the host. Hydroxyapatite [HAP: Ca_10_(PO_4_)_6_(OH)_2_] is a bioactive substance that can be found from a natural or synthetic source. It is considered as a good bone graft material because of its excellent biocompatibility, rigidity, nonimmunogenicity, and also its crystallographic structure is the same as the bone mineral [3, 7]. Constant research are concentrated on polymer/ceramic-based scaffolds to fix up the human skeleton, some of which are HAP/polymer [7], HAP/collagen [8–9], HAP/chitosan [10], HAP/collagen/poly (lactic acid) [11], HAP/alginate/collagen [12] and HAP/gelatin [13–15]. HAP is brittle. But the biocomposite of nanohydroxyapatite crystal with organic collagen matrix form strong and flexible composites. It shows remarkable biological and mechanical supports in clinical sectors [3–4]. Though HAP/collagen composite scaffold confer excellent properties in the strength and toughness of the bone [4, 5], the cost and source of the collagen impede the review of the process [7]. However, the combination of HAP/chitosangelatin has received more attention in this regard. A highly porous structure affects the development of loadbearing scaffolds [6]. Some research claims that the bone graft material having the pore size in the range of 100–500μm helps to grow new bone cells successfully [8–12, 16–17]. Gelatin, a natural polymer, can control the pore size to the optimal level. It can be derived from collagen which exhibits the potential advantage for cell adhesion [4,18]. The uniform dispersion of HAP nanoparticles over the HAP/chitosan-gelatin composite scaffold largely depends on the gelatin matrix. It acts as a binding agent to promote the bonding between particle and polymer matrix. [5–6]. To evaluate high compressive strength, scientists consider chitosan as a novel biomaterial. It shows a larger degradation rate by the enzymes remaining in the human body than the bioceramics [7,19]. The crystallinity of calcium phosphate is not affected by chitosan. It accelerates bone regeneration by offering a surface that deals with the growth factor, receptors, and adhesion protein [6]. It possesses an excellent quality of wound healing and serves as nontoxic, hemostatic, and biocompatible material [17]. Though it shows poor mechanical strength itself, the combination of chitosan with other materials like hydroxyapatite and gelatin promotes to overcome the unexpected properties [20]. A suitable crosslinker glutaraldehyde has been used to control the biodegradation rate which can bond with some relevant functional groups to make the scaffold mechanically strong.

As a single component like HAP or gelatin cannot mimic the cellular growth of ECM, the bioceramic nanoparticles along with the biopolymer matrix generate a good mechanical strength as well as naturalize cell adhesion, proliferation, and disintegration [3, 18]. To our knowledge, no study has reached complete success to replace the bone. We are intending to optimize the HAP ratio into the composition to fabricate a HAP/chitosangelatin composite for the development of bioactive bone scaffold. HAP/chitosan-gelatin composite has been prepared by using a wet chemical precipitation method where glutaraldehyde is used to form intermolecular crosslinks between protein molecules. The prepared scaffolds were characterized for cytotoxicity, biodegradability, and in vitro biocompatibility, physical, chemical, and morphological properties. This investigation may add a successful contribution to the development of superior scaffolds for bone tissue engineering.

## 2. Materials and methods

Chitosan and gelatin were purchased from Sigma-Aldrich Chemie GmbH (Taufkirchen, Germany) and Biochemical (England), respectively. The starting materials [Ca(NO_3_)_2_.4H_2_O] and [(NH_4_)_2_HPO_4_] were bought from Merck Ltd. (Mumbai, Maharashtra, India) and Loba Chemie Pvt. Ltd. (Mumbai, Maharashtra, India) respectively. The NaOH pellets and the glutaraldehyde solution (25% solution in water) were obtained through Merck Ltd. All of these chemicals were used as received without further purification.

### 2.1. Preparation of Hydroxyapatite [Ca_10_(PO_4_)_6_(OH)_2_] nanoparticle

**Figure 1:**
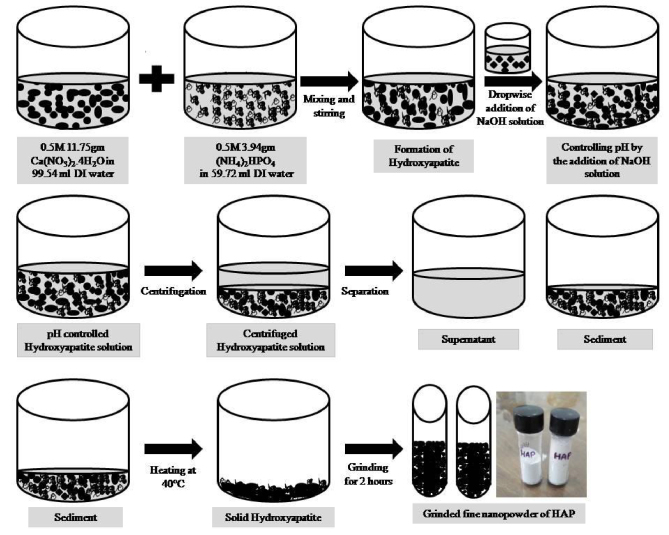
Schematic diagram of preparing hydroxyapatite nanoparticle.

Calcium nitrate tetrahydrate [Ca(NO_3_)_2_.4H_2_O] and di-ammonium hydrogen phosphate [(NH_4_)_2_HPO_4_] were chosen as precursor substances to fabricate hydroxyapatite [Ca_10_(PO_4_)_6_(OH)_2_] nanoparticle. Sodium hydroxide (NaOH) solution was taken as a pH controller of the solution. The reaction was conducted at room temperature. The procedure started by weighing the required salts according to the molar ratio of hydroxyapatite which is 1.67. The solutions of 0.5 M of [Ca(NO_3_)_2_.4H_2_O] and 0.5 M of [(NH_4_)_2_HPO_4_] were prepared by mixing the corresponding salts with deionized (DI) distilled water. In this case, 3M of NaOH was used as a coprecipitation agent. The NaOH solution was mixed dropwise to control the pH. The solution was then followed by centrifugation and the particles were washed 10 times to remove the additional NaOH solution. Heat was applied to the solution up to 40 °C with simultaneous stirring by a magnetic stirrer. The dried [Ca_10_(PO_4_)_6_(OH)_2_] was ground using mortar and pestle to form fine, smooth, and average-sized hydroxyapatite nanopowder. It is significant to note that, a total of 5 g of hydroxyapatite powder was prepared in each time. Figure 1 corresponds to the flow chart of hydroxyapatite preparation.

The following reaction was used to prepare Ca_10_(PO_4_)_6_(OH)_2_:

10Ca(NO_3_)_2_.4H_2_O + 6(NH_4_)_2_HPO_4_ + 2H_2_O = Ca_10_(PO_4_)_6_(OH)_2_+ 12NH_4_NO_3_ + 8HNO_3_

### 2.2. Preparation of Hydroxyapatite/Chitosan-gelatin scaffold

The expected properties of the prepared bone scaffolds differ greatly depending on methods. In this study, six different kinds of scaffolds were prepared by varying the concentration of hydroxyapatite, gelatin, chitosan, and glutaraldehyde solutions. The chitosan solution was prepared by dissolving the chitosan powder in the solution of acetic acid and DI distilled water. The prepared solution was continuously stirred at room temperature for 24 h. When the chitosan was dissolved homogeneously, it was preserved in a beaker. The different weight percentages of glutaraldehyde solutions like 0.2% and 2.0% were prepared by dissolving glutaraldehyde (50%) in DI distilled water. The solution of hydroxyapatite was prepared by dissolving 0.2 g hydroxyapatite nanopowder in 66.67 mL DI distilled water. The hydroxyapatite solution was then ultra-sonicated so that the HAP nanopowder could thoroughly disperse in the water. The ratio of preparing hydroxyapatite solution was maintained for all the cases. The ultrasonicated solution was kept overnight so that hydroxyapatite could precipitate in the beaker. The hydroxyapatite paste was obtained after separating the supernatant from the beaker.

To prepare the scaffold, hydroxyapatite paste was mingled with chitosan solution under continuous stirring. Then the gelatin powder was added to the mixture under agitation so that the gelatin powder could homogeneously mix with the HAP/chitosan mixture. The prepared glutaraldehyde solution was added dropwise to the solution as a crosslinking agent. The final solution was poured into a cylindrical die for mold casting and kept it rest for 24 h to obtain the shape. Table 1 corresponds to the composition of the prepared bone scaffolds.

**Table 1:** Composition of the prepared bone scaffolds.

No. of processes	HAP Powder		Chitosan solution (g)	Gelatin (g)	Glutaraldehyde solution (2% and 0.2%) (g)	Sample designation
	Mass ratio of HAP (%)	HAP content (g)				
Process-1	0	0.0	14.33	1.0	0.7	Scaffold-1
Process-2	10	0.2	12.90	0.9	0.7	Scaffold-2
Process-3	20	0.4	11.47	0.8	0.7	Scaffold-3
Process-4	30	0.6	10.03	0.7	0.7	Scaffold-4
Process-5	40	0.8	8.60	0.6	0.6	Scaffold-5
Process-6	50	1.0	7.16	0.5	0.5	Scaffold-6

The crosslinking reactions of Glutaraldehyde (GA) with chitosan (CS) and gelatin (Glt) are shown below where [|CHO] and [|C=N|] indicates the aldehyde group and imine group respectively.

GA|CHO + CS|NH_2_GA|C=N|CS

GA|CHO + Glt|NH_2_GA|C=N|Glt

**Figure 2:**
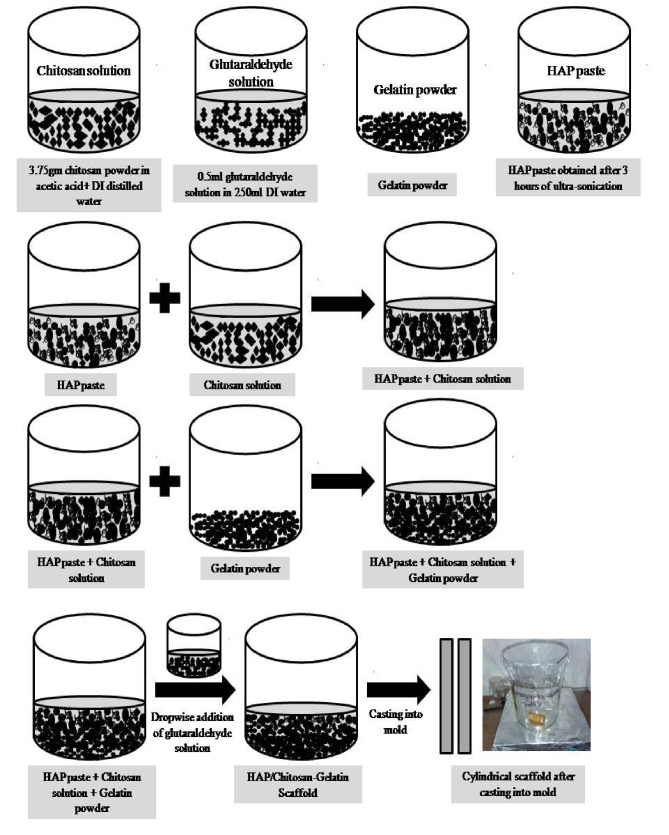
Schematic diagram of the preparation of HAP/chitosan-gelatin scaffold.

Figure 2 shows the procedure of preparation of HAP/chitosan-gelatin scaffolds. Here, both the 0.2 wt% glutaraldehyde solutions and the 2.0 wt% glutaraldehyde solutions were added to examine the toxic effect of the above scaffolds. The scaffolds prepared by this method were air-dried at ambient temperature at 25 ◦ C for 3 weeks so that the water could be removed entirely. The scaffolds were preserved by wrapping them with foil papers.

## 3. Results

### 3.1. X-ray Diffraction (XRD)

**Figure 3:**
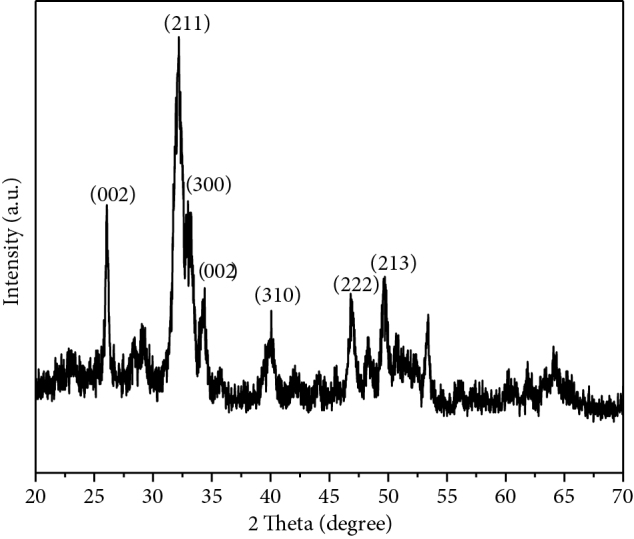
X-ray diffraction pattern of hydroxyapatite.

X-ray Diffraction has been performed over hydroxyapatite [Ca_10_(PO_4_)_6_(OH)_2_] nanopowder to analyze the structural properties. The pattern of this study confirms the successful formation of hydroxyapatite and matched with the HAP standard JCPDS pattern (card number 9-0432) shown in Figure 3 [21]. The spectra reveal the polycrystalline structure of hydroxyapatite. The characteristic planes for hydroxyapatite are generally found as (211), (112), and (300). However, in this case, the (112) plane cannot be detected in the XRD pattern of as-prepared hydroxyapatite due to its poor crystallinity. The poorly crystalline hydroxyapatite becomes highly crystalline when the temperature is increased to a certain temperature [22].

The Miller indices (hkl) of the diffraction peaks calculated from Figure 3 are referred to as the hexagonal axes. The lattice parameters, crystallite size, crystallinity and porosity of hydroxyapatite nanoparticles have been calculated using the strong peaks (211), (300), (002) and (310) and listed in Table 2. Brag’s diffraction law and crystal geometry equation have been used to calculate the lattice parameters. The crystallite size has been measured by the Debye-Scherrer equation. Crystallinity has been obtained by dividing the total area of crystalline peaks by the total area under the diffraction curve (crystalline plus amorphous peaks). The porosity (%) has been calculated by dividing the volume of voids (total volume - volume of the solid) by the total volume of the sample. The calculated porosity of hydroxyapatite is about 67.45% which has a large impact to bind up the network composite. It supports interlocking mechanically, which helps firm fixation with the other composite materials [23]. The X-ray Diffraction has also been operated on the prepared network composite to study the comparative crystallinity.

**Table 2:** The lattice parameters a and c, crystallite size, crystallinity, and porosity of hydroxyapatite nanoparticles.

Name of the material	a (Å)	c (Å)	Crystallite size (nm)	Crystallinity X_c_ (%)	Porosity (%)
Hydroxyapatite	9.359	6.849	30.03	47.64	67.45

**Figure 4:**
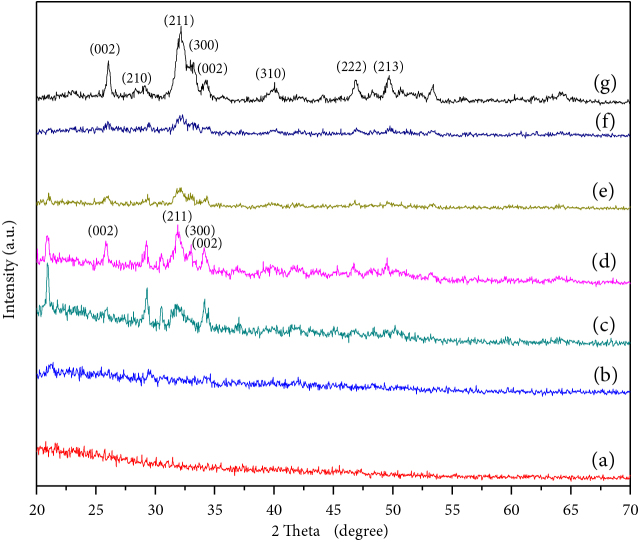
X-ray diffraction patterns of composite network with HAP content (a) 0%, (b) 10%, (c) 20%, (d) 30%, (e) 40%, (f) 50%, (g) 100%.

Figure 4 shows the XRD patterns of the composite scaffolds which shows that the peak starts to split into many smaller peaks with increasing the amount of hydroxyapatite. The degree of crystallinity or structural order of composites is shown in Table 3.

**Table 3:** The crystallinity of the composite network with different amounts of HAP content.

Amount of HAP content in composite	Composite with 20% HAP content	Composite with 30% HAP content	Composite with 40% HAP content	Composite with 50% HAP content	100% HAP content
Crystallinity X_c_ (%)	31.74	43.81	29.51	28.53	47.64

The crystallinity of the composite network varies with the incorporation of different amounts of HAP. No peak is observed for 0% and 10% HAP content as shown in Figures 4a–4b. Figure 4c shows that peaks arise with 20% HAP content. XRD pattern of 30%, 40%, 50% and 100% HAP content shows crystallinity as shown in Figures 4e–4g. At 30% of HAP content, crystallinity shows the highest value compared to others. In this case, the XRD pattern of composite scaffold complies with the XRD pattern of hydroxyapatite, and the characteristics peaks of hydroxyapatite become apparent. This result suggests us to consider scaffold-4 (with 30% HAP content) as an optimum scaffold.

### 3.2. Transmission electron microscope (TEM) analyses

**Figure 5:**
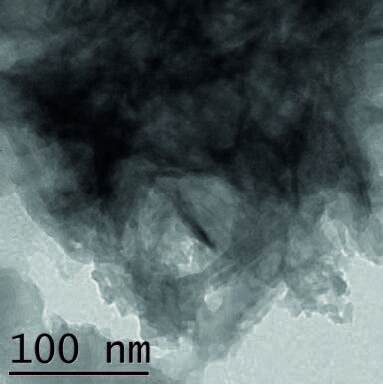

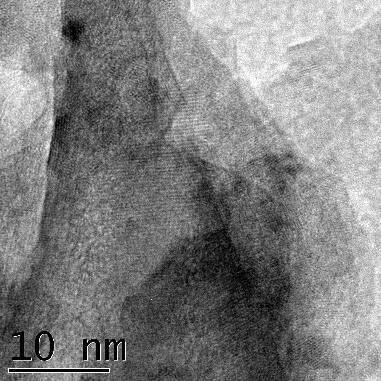

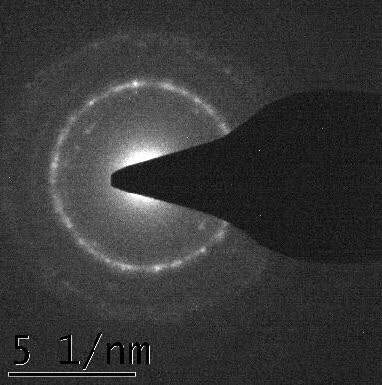
(a) TEM image of as-prepared hydroxyapatite powder, (b) HRTEM image of hydroxyapatite powder, (c) Polycrystalline ring SAD pattern with hydroxyapatite interplanar spacing.

The as-prepared hydroxyapatite powder has been examined by a Transmission Electron Microscope (TEM) to investigate the morphology and shape of the individual particles. It is revealed from Figure 5a that the crystal of hydroxyapatite nanopowder has a needle-like shape which appears to agglomerate and showing interconnected and porous structures. It is obtained that the HAP particles have a length of 100 nm and a diameter of around 2 nm. The TEM image of the needle-like crystal structure of the as-prepared hydroxyapatite indicated that the particles may become regular and smooth with increasing ripening time [24, 25].

Figure 5b shows the high-resolution transmission electron microscope (HRTEM) image which confirms the crystallinity of the corresponding material. The TEM results reveal the oriented nucleation and homoepitaxial growth of the assembled hydroxyapatite. Figure 5c shows the selected area diffraction (SAD) pattern with a polycrystalline ring which corresponds to HAP hexagonal phases. The first ring corresponded to the (002) plane which indicates that HAP grows along the (002) planes. The next strong diffraction ring corresponds to the (211) plane. The (112) plane and the (300) plane are quite close to the (211) plane. The diffraction ring of HAP is found very clear and sharp, displaying a higher crystallinity, which is consistent with the XRD results.

### 3.3. Scanning electron microscope (SEM) analysis

**Figure 6:**
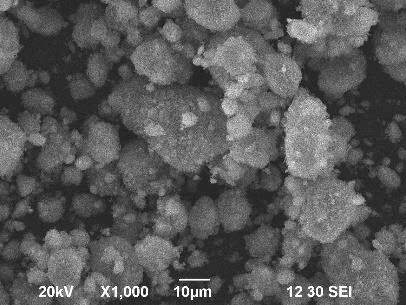

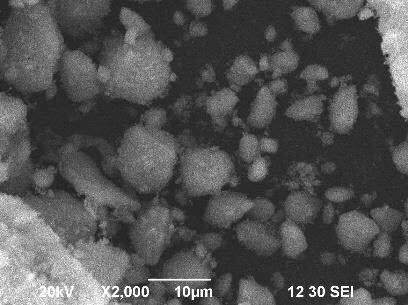

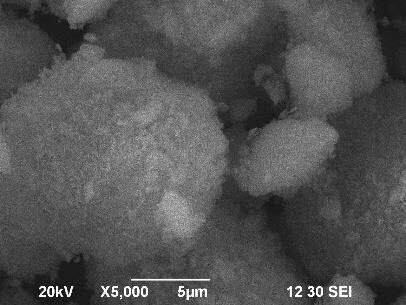
SEM micrographs of as-prepared hydroxyapatite sample: (a) at 1000×, (b) at 2000×, (c) at 5000× magnification.

**Figure 7:**
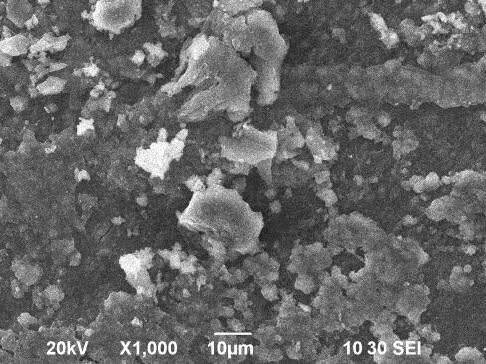

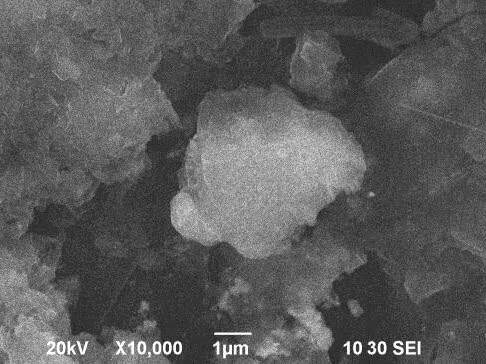

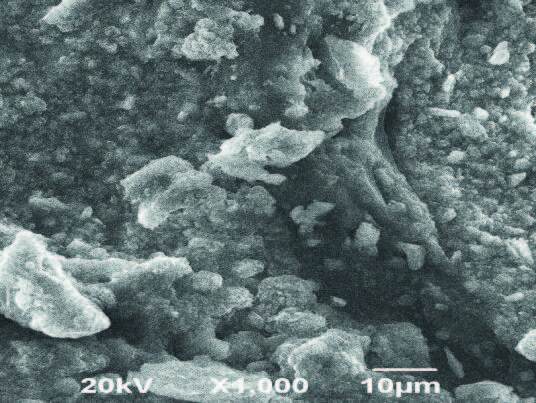

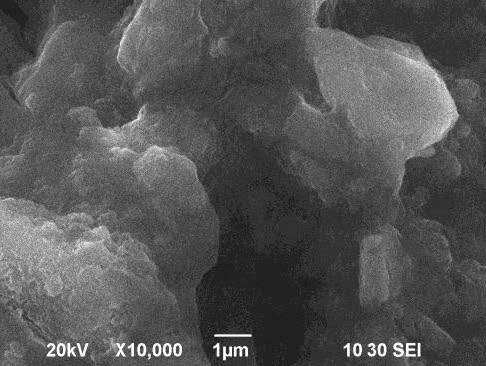
SEM micrographs of composite network with HAP content at (i) 1000× magnification, (ii) 10000× magnification of (a) 30%, (b) 50%.

The morphology of the hydroxyapatite has been observed by Scanning Electron Microscope (SEM). The grain size of hydroxyapatite has been calculated by a scaling method from the SEM images Figures 6a–6c at different magnifications. The average size of aggregated hydroxyapatite spherical chunks with smaller individual particles is calculated at about 7.07 μm. Figures 7a and 7b shows the SEM images of network composites with 30% and 50% HAP content at different magnifications.

**Figure 8:**
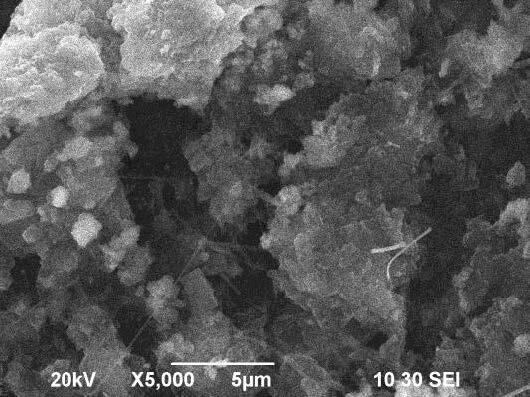

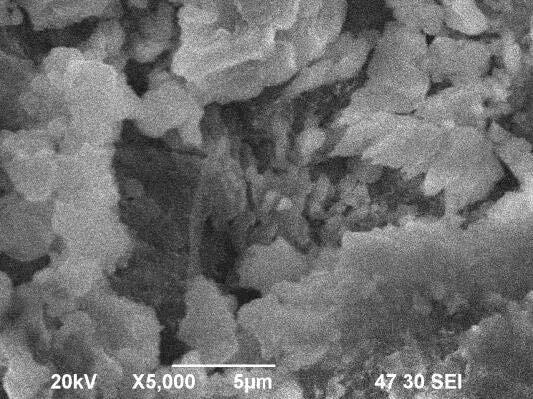
SEM micrographs of (a) prepared network composite (with 30% HAP content), (b) human bone at 5000× magnification.

SEM images of prepared scaffold and human bone (phalanges) are shown in Figures 8a and 8b. A large number of pores have been observed in the scaffold images. The pore size has been calculated by a scaling method and it is found to vary between 20–50 μm where extremely small pores have been ignored.

The range of the size of the pores must be larger than 100–500 μm to colonize the pores by bone tissue. Generally, a scaffold can be described by its average pore size, pore interconnectivity, and pore shape. These pores offer an ideal environment for the attachment and proliferation of the bone cells. According to this study, the pore size of our sample is slightly less than the ideal one. The pore size can be controlled by changing the concentration of hydroxyapatite or gelatin. The more the gelatin is added, the more electrostatic interaction between hydroxyapatite and gelatin will occur which results in the decrease in pore size [26]. We are trying to increase the pore sizes by using a balanced amount of hydroxyapatite and gelatin.

#### 3.4. Functional group analysis

**Figure 9:**
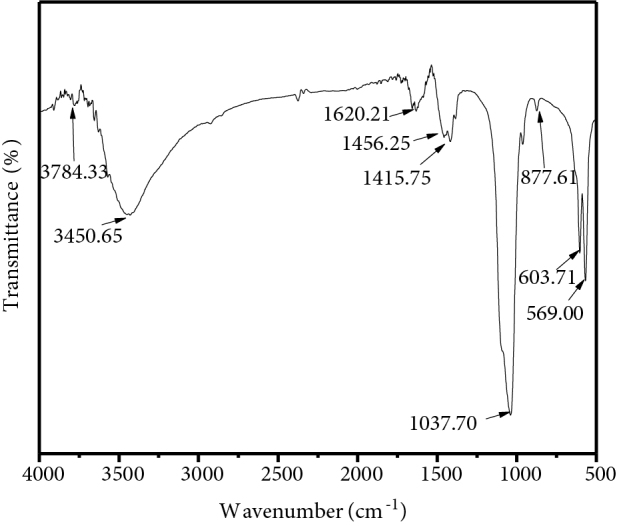
Fourier transform infrared spectrum of as-prepared hydroxyapatite.

Fourier transform infrared spectroscopy (FTIR) analysis has been performed over prepared hydroxyapatite powder to analyze the presence of different functional groups shown in Figure 9. The appropriate crystallographic phase formation is determined by identifying various functional groups in synthesized samples. In the FTIR spectrum, different bands represented different groups. The bands at around 3784.33 cm^-1^ and 603.71 cm^-1^ indicate the stretching and vibrational or bending mode of vibration respectively of the apatitic hydroxyl group (OH^-^) in the hydroxyapatite crystal. A broad peak around 3450.65 cm^-1^ wavenumbers is referred to as the stretching mode of the hydroxyl group. The peak at 1620.21 cm^-1^ is assigned to the bending of hydroxylion due to the chemical absorbance of H_2_O. The FTIR spectra of hydroxyapatite nanopowder fabricated by wet chemical precipitation technique are approximately matched with the FTIR spectra of our prepared sample [27, 28]. Table 4 shows the functional group elements formed in as-prepared hydroxyapatite nanoparticles.

**Table 4:** Functional group elements of synthesized as-prepared hydroxyapatite nanoparticles.

Wavenumber of the corresponding functional group (cm−1)	Stretching or bending mode	Functional group
3784.33	Stretching mode	OH^-^
603.71	Bending mode	OH^-^
3450.65	Stretching mode	OH^-^ due to absorbed H_2_O
1620.21	Bending mode	OH^-^ due to absorbed H_2_O
1037.70	Asymmetric stretching	PO^3-^_4_
569.0	Asymmetric bending	PO^3-^_4_
877.61	Out of plane bending	CO^2-^_3_

**Figure 10:**
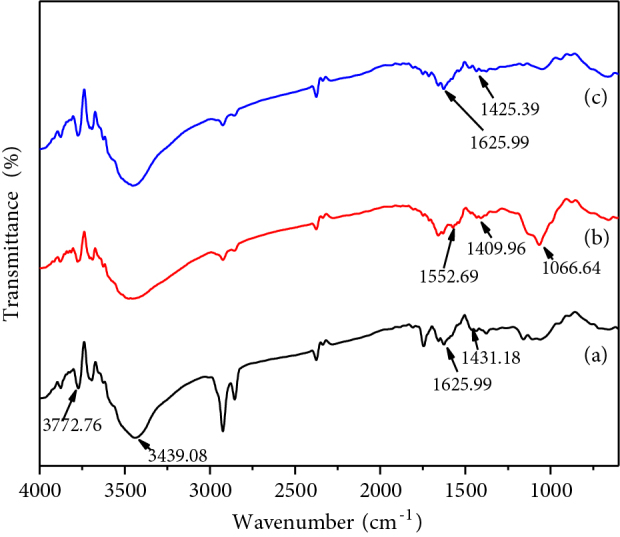
ATR-FTIR of (a) chitosan powder, (b) HAP/chitosan-gelatin network, (c) gelatin powder.

The ATR-FTIR spectra of chitosan powder, HAP/chitosan-gelatin network, and gelatin powder are shown in Figures 10a–10c. The peak for amide I bands at 1625.99 cm−1 is present only in the chitosan and gelatin which has become disappeared in the composite network. In this case, the imine (C=N) group has formed due to the Schiff base reaction occurred between the amino groups from chitosan and gelatin and the aldehyde groups from the glutaraldehyde [29]. The bands at 1431.18 cm−1 (Figure 10a) indicate the amide II bands for chitosan. It is noticed from Figure 10b that the peak arises at 1409.96 cm−1 which might be attributed to the mode superposition of the hydroxyapatite OH group and the chitosan amide II groups [27,28]. The absorption peak at 1066.64 cm−1 in the composite network (Figure 10b) corresponds to the phosphate vibration. The significant bands at 1552.69 cm−1 confirm the presence of the imine (C=N) group [3]. Figure 10c also shows the peaks at 1625.99 cm−1 and 1425.39 cm−1 which corresponds to the amide I and amide II bands for gelatin.

#### 3.5. Thermo-gravimetric analysis

**Figure 11:**
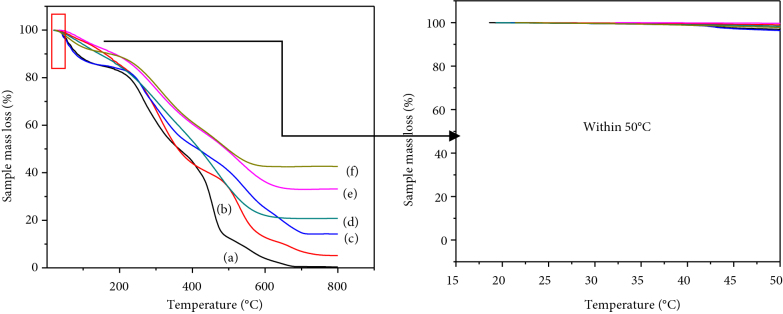
(i) Thermo-gravimetric analysis of composite network with HAP content (a) 0%, (b) 10%, (c) 20%, (d) 30%, (e) 40%, (f) 50% , (ii) close snap of TGA graphs from 0–50 °C.

It is seen from Figure 11 that there is a weight loss of 10 wt% below 150 °C owing to the removal of a water molecule from the composite upon heat treatment [26]. The composite without HAP content (Figure 11a) vanishes above 600 °C. The composites have remained 5.2%, 14.24%, and 20.82% at the temperature above 800 °C for scaffolds with 10%, 20%, and 30% HAP content respectively (Figures 11b–11d). But the scaffolds with 40% and 50% hap content have remained 36.66% and 42.67% after the same amount of heat treatment (Figures 11e and 11f). The endothermal curve just below 800 °C indicates the release of carbon dioxide (CO_2_) from the composite which is mixed with hydroxyapatite during the co-precipitation method [30]. It is also noticed that weight loss has become negligible after 785 °C. Beyond 785 °C, no significant weight loss and an almost stable curve confirm the thermal stability of HA powder. The compositional analysis of the thermogravimetric data is depicted in Table 5.

**Table 5:** A comparative compositional analysis of thermo-gravimetric data.

Presence of HAP content in composite network	Composite without HAP content	Composite with 10% HAP content	Composite with 20% HAP content	Composite with 30% HAP content	Composite with 40% HAP content	Composite with 50% HAP content
Remaining amount of composite after 800 °C (%)	0.0	5.2%	14.24%	20.82%	36.66%	42.67%

The temperature-dependent behavior can be interpreted by the intermolecular interaction among the molecules of the composite scaffolds because the composites get more compacted by increasing HAP content. The interaction between the amino groups and PO^3-^_4_ is weaker than the interaction between Ca^2+^ from hydroxyapatite and COO^-^ from gelatin which helps the formation of hydroxyapatite in the network composites. Therefore, it can be considered that the carboxyl group has a stronger effect on the crystallinity of hydroxyapatite on the surface of the scaffolds [31].

#### 3.6. Evaluating cytotoxicity: qualitative examination by VERO cell

**Figure 12:**
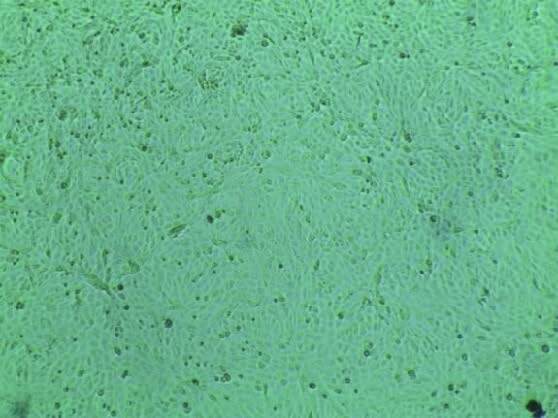

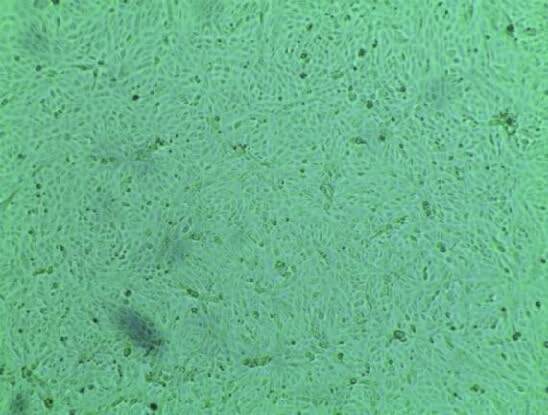
Microscopic images of (a) Vero cell, (b) Vero cell with control solution (untreated cell without composite scaffold).

The preparation of HAP/chitosan-gelatin scaffolds includes the addition of glutaraldehyde solution (wt%) to increase the stability of gelatin in water. Glutaraldehyde is cytotoxic itself. To examine the toxic level of glutaraldehyde, different samples have been prepared by varying the concentration of glutaraldehyde. Then the prepared scaffolds have been performed with Vero cell (a normal animal cell) to study the cytotoxicity effect quantitatively. The solution is considered as a positive control solution in the absence of network composite which has also been studied. An inverted light microscope has been used to observe the general morphological changes of VERO cells. The microscopic images of the Vero cells with medium and with control solution are shown in Figures 12a and 12b.

**Figure 13:**
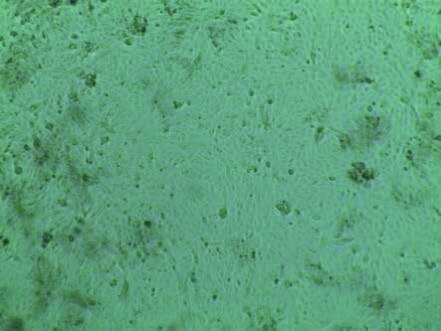

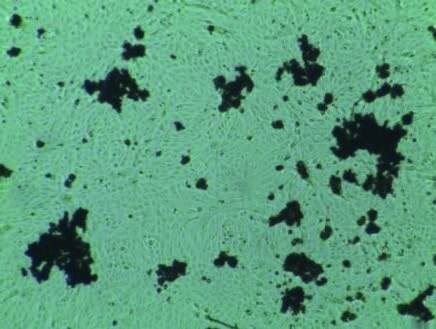

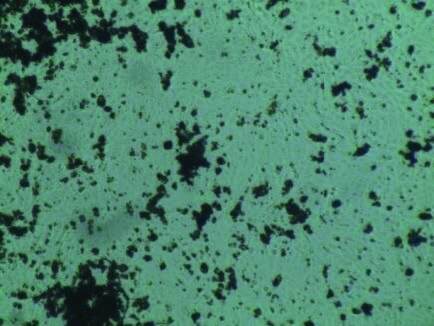
Microscopic images of Vero cells with composite scaffold solution of concentration of (a) 1 mg/mL with 0.2 wt%, (b) 0.5 mg/mL with 2 wt%, (c) 1 mg/mL with 2 wt% glutaraldehyde solution (gluta).

Figure 13 shows the cell viability with membrane and shape integrity similar to the cells in the control solution after 48 h of incubation with the network scaffold of different concentrations.

It is clear from Figures 12a-12b and Figures 13a–13c that both the control and composite scaffold has no remarkable change in the cellular apoptotic activity for 48 h of incubation time. From Figures 13b–13c, it is noticed that the particles have agglomerated on the cell which might be attributed to the higher concentration of the solution. Even ßthough the concentration of glutaraldehyde (wt%) has increased, the survival of cells remains almost the same as the control solution. As a consequence, it is conjectured that the scaffolds with 0.2–2 wt% glutaraldehyde solutions have no toxic effect upon the Vero cell line.

#### 3.7. Quantitative examination by MTT-assay

**Figure 14:**
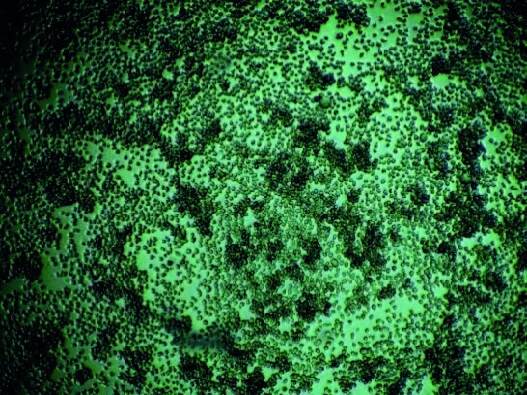

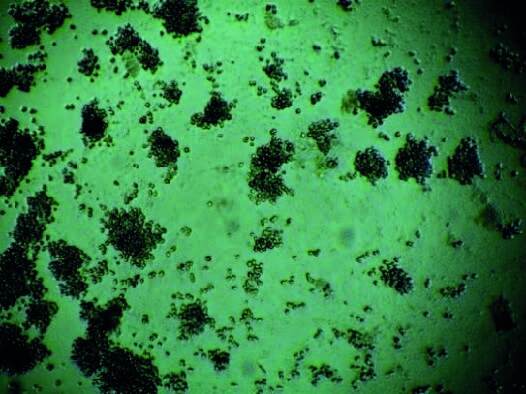

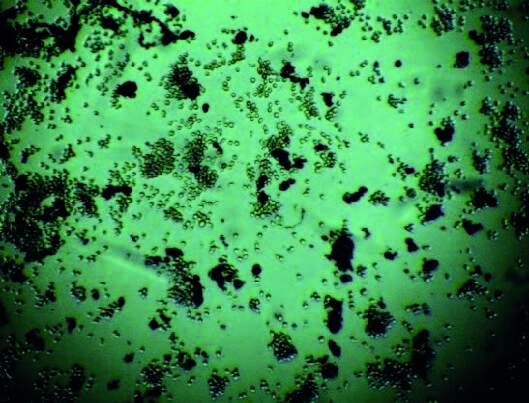

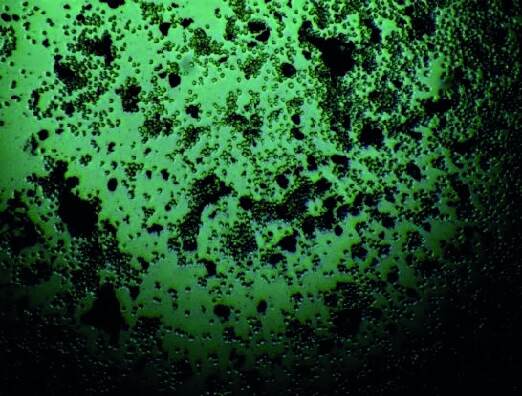
Microscopic images of Molt-4 cells with composite scaffold solution of concentration of (a) control solution, (b) 1 mg/mL with 0.2 wt%, (c) 0.5 mg/mL with 2 wt%, (d) 1 mg/mL with 2 wt% glutaraldehyde solution (gluta).

To test the toxic effect quantitatively, the Molt-4 cells’ viability has been examined on the scaffold by tetrazoliumbased assay (MTT test). Figure 14 shows the Molt-4 cells in the control solution and scaffolds with different concentrations. Table 6 reveals that the cells’ viability in the control solution does not show any toxicity (Figure 14a). Figures 14b and 14c shows that the viability of Molt-4 cells remains almost the same but significant change is observed in the case of Figure 14d.

**Table 6:** Evaluation of toxicity of bone replacing scaffolds on Molt-4 cell line.

Sample ID	Survival of Molt-4 cells	Remarks
Control solution	100	A little toxicity has been observed for Scaffolds with 1 mg/mL with 2 wt% glutaraldehyde on Molt-4 cell line
Scaffolds with 1 mg/mL with 0.2 wt% glutaraldehyde solution	98.21	
Scaffolds with 0.5 mg/mL with 2.0 wt% glutaraldehyde solution	96.91	
Scaffolds with 1 mg/mL with 2.0 wt% glutaraldehyde solution	78.93	

**Figure 15:**
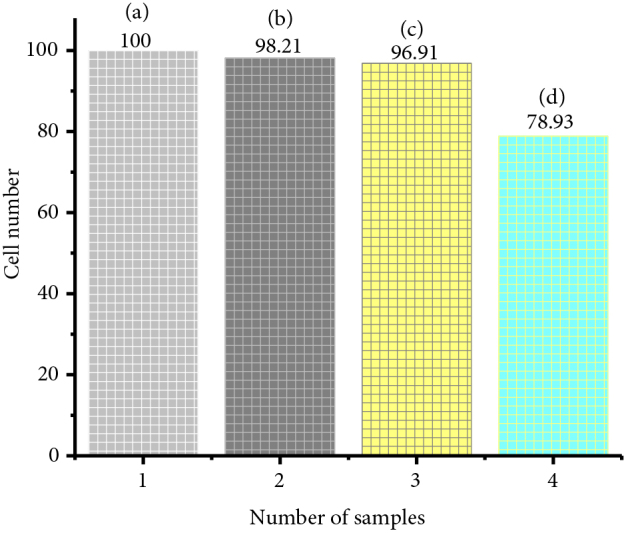
Schematic diagram of in vitro cell viability assay of Molt-4 cells for (a) control solution, (b) 1 mg/mL with 0.2 wt%, (b) 0.5 mg/mL with 2 wt%, (c) 1 mg/mL with 2 wt% glutaraldehyde solution.

The cell viability of prepared composites has been examined by performing the samples in both the animal cell (VERO cell) and the human blood cell (Molt-4). As glutaraldehyde has been used as a crosslinker, the toxicity has evaluated by changing the concentration of glutaraldehyde (wt%). Figures 15a–15d show that the cytotoxic effect of the scaffolds increases with increasing the weight percentage of the glutaraldehyde solution. The scaffold with 0.2 wt% glutaraldehyde solutions seems to have the least toxic effect on the cell survival compared to the control solution (solution without composite sample). This suggests that it is good enough to keep the concentration of glutaraldehyde solution below 0.2 wt%.

A biocompatible product that has high purity and a significant amount of porosity are required in a scaffold and bone grafts to accommodate fluid or cell transfer and tissue ingrowth. The structural properties of hydroxyapatite nanoparticles are attractive for bone tissue engineering, bone void fillers, implant materials as a form of bone grafts or scaffold, and coating material on the implant of metal composites. The result achieved from this study demonstrates that a proper bone replacing scaffold can be made up by controlling the amount of hydroxyapatite, gelatin, and chitosan which will be biocompatible, biodegradable, and bio-friendly for any living organism.

### 4. Conclusion

The progress of the scaffolds with different concentrations of the constituents including hydroxyapatite has been focused in this study. Hydroxyapatite nanopowder with crystallite size 30.03 nm has been successfully synthesized by wet chemical precipitation method. The diffraction ring found from the Transmission electron microscope (TEM) shows a higher crystallinity of HAP crystal. A scanning electron microscope (SEM) reveals that the nanopowders are distributed in the aggregated form. The ATR-FTIR spectra confirm the formation of bone replacing scaffolds by showing different band groups like PO3−4 , C=O, | CH2 , | C=N| , etc., which approximately matches with the human bone (phalanges). The ATR spectra of the prepared scaffold show similarity with the ATR spectra of human bone. In the case of a qualitative study, Vero cells have been used which shows approximately zero toxic effect over the scaffolds. The MTT-assay suggests that a proper balance between the cell viability and gelatin stability can only be maintained by keeping the weight percentage of glutaraldehyde solution lower than 2%. The prepared composite networks with appropriate mechanical strength will offer good potential in case of bone substitution and any other biomedical application regarding osteoconductivity and biodegradability. The effective feedback leading to these confirmations will increase the curiosity of the bone replacing scaffolds from the laboratory to practical biomedical applications.
